# Beyond Human Perception: Sexual Dimorphism in Hand and Wrist Radiographs Is Discernible by a Deep Learning Model

**DOI:** 10.1007/s10278-018-0148-x

**Published:** 2018-11-26

**Authors:** Sehyo Yune, Hyunkwang Lee, Myeongchan Kim, Shahein H. Tajmir, Michael S. Gee, Synho Do

**Affiliations:** Department of Radiology, Massachusetts General Hospital, 25 New Chardon Street Suite 400B, Boston, MA 02114 USA

**Keywords:** Machine learning, Artificial intelligence, Sexual dimorphism, Sexual development, Bone development

## Abstract

Despite the well-established impact of sex and sex hormones on bone structure and density, there has been limited description of sexual dimorphism in the hand and wrist in the literature. We developed a deep convolutional neural network (CNN) model to predict sex based on hand radiographs of children and adults aged between 5 and 70 years. Of the 1531 radiographs tested, the algorithm predicted sex correctly in 95.9% (*κ* = 0.92) of the cases. Two human radiologists achieved 58% (*κ* = 0.15) and 46% (*κ* = − 0.07) accuracy. The class activation maps (CAM) showed that the model mostly focused on the 2nd and 3rd metacarpal base or thumb sesamoid in women, and distal radioulnar joint, distal radial physis and epiphysis, or 3rd metacarpophalangeal joint in men. The radiologists reviewed 70 cases (35 females and 35 males) labeled with sex along with heat maps generated by CAM, but they could not find any patterns that distinguish the two sexes. A small sample of patients (*n* = 44) with sexual developmental disorders or transgender identity was selected for a preliminary exploration of application of the model. The model prediction agreed with phenotypic sex in only 77.8% (*κ* = 0.54) of these cases. To the best of our knowledge, this is the first study that demonstrated a machine learning model to perform a task in which human experts could not fulfill.

## Introduction

A picture may be worth a thousand words, but describing it should be much more concise. When radiologists interpret medical images, they look for specific features associated with disease and may overlook certain features, either intentionally or unconsciously, that are not obviously indicative of pathology. This practice leads to more efficient image interpretation; however, there are likely to be additional clinically relevant imaging features that are beyond current human radiologist visual discernment.

Computational models that used machine learning, especially deep learning, has shown remarkable performance in medical image analysis over the past few years in a variety of tasks including classifying skin cancer [1] and predicting cardiovascular risk [2]. However, most of the studies that leverage deep learning for medical image analysis focus on a replicating a task already performed by humans. In addition to mimicking humans, machine learning also offers the potential of identifying significant imaging features that are beyond a radiologist’s visual search pattern, and perhaps enhance the diagnostic utility of medical images.

Hand radiographs are widely used for assessment of skeletal age because the examination is straightforward to perform with minimal radiation exposure and include multiple bones in a single view. Bone age assessment (BAA) is used to find abnormalities in skeletal development, monitoring growth hormone therapy, diagnosis of endocrine disorders, predicting adult height, and planning surgery of the long bones or vertebral column [3]. Many children with precocious puberty, absent secondary sexual characteristics, or short stature undergo BAA, as bone maturation is closely related to sexual development. In this subset of patients for whom hormone replacement treatment, future fertility, and psychosocial support are important issues, information about sexual development will add important value to this simple imaging process.

To the best of our knowledge, there has not been reported tools or parameters by which sex can be reliably identified from hand radiographs in children. Because a patient’s sex is provided for radiologists to interpret bone age radiographs, there is no established set of sex-specific radiographic features. In this study, we developed a deep learning system that analyzes hand radiographs of individuals of 5 to 70 years of age and predicts sex. Two radiologists reviewed the system output to describe the patterns used to distinguish sexes. To find at which age it becomes evident, we analyzed the system output stratified by age. In addition, we tested the system on separately selected radiographs to explore how the deep learning–based sexual dimorphism is expressed in people with special conditions.

## Materials and Methods

### Study Subjects

The institutional review board of the Massachusetts General Hospital approved the study protocol and waived the requirement for informed consent based on the substantial difficulty to acquire consent and the minimal risk to the study subjects. All study data were retrieved from an institutional registry which only includes data from patients who agreed with use of their data for research purposes. Previously identified pediatric left-hand radiographs of 4278 females and 4047 males were first included in the current study data [4]. Additionally, left-hand radiographs of male and female adults of age 19 or older were collected from the institutional research database. Among all left-hand radiographs in the database, we found 2282 cases that are reported as normal in radiology reports. In total, we compiled a dataset of 10,607 (5459 females and 5148 males) radiographs of the left hand and wrist from patients aged 5 years to 70 years.

**Fig. 1:**
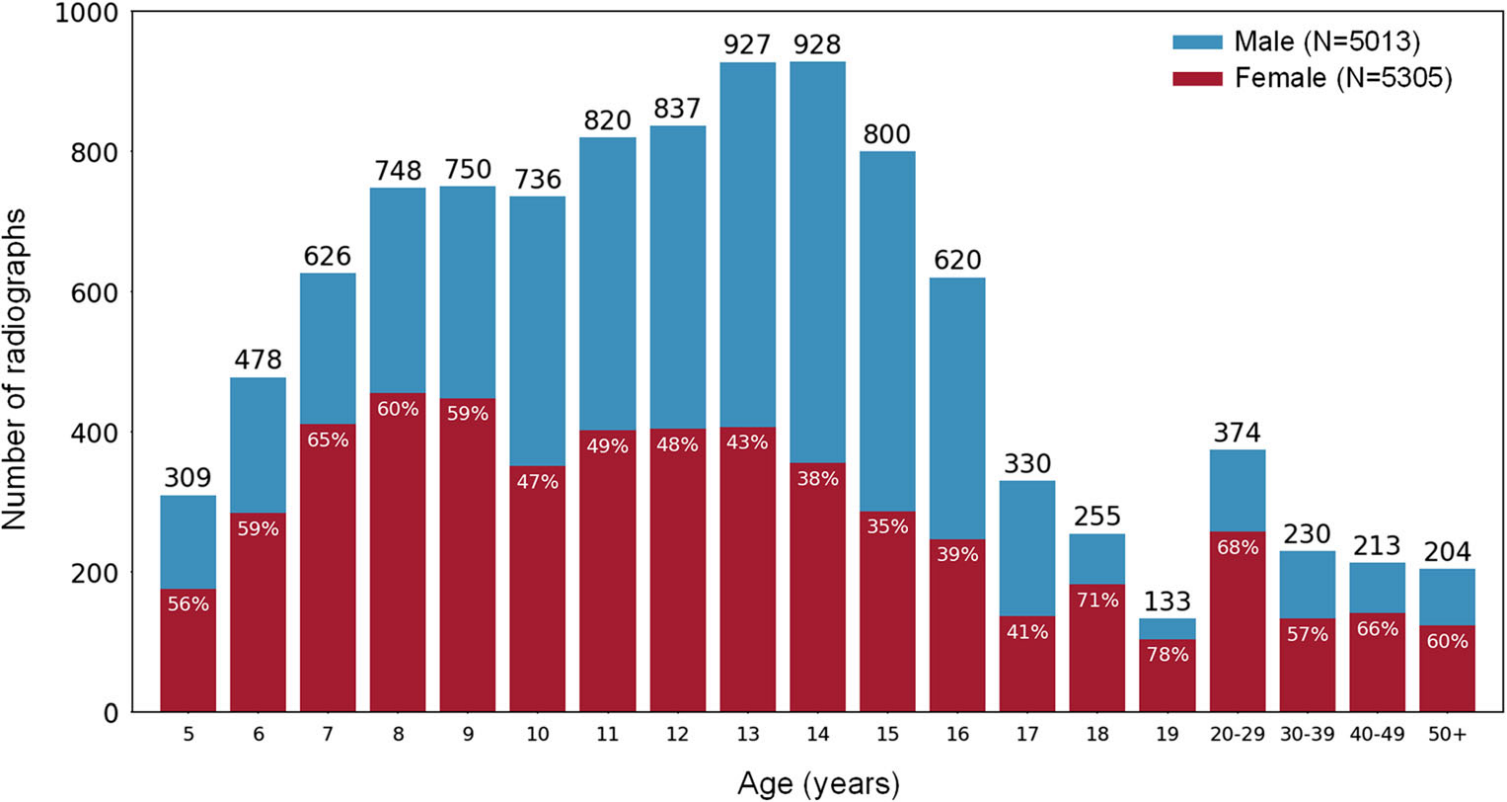
Age and sex distribution of the study subjects included in the final dataset. The numbers on top of each bar indicates the number of radiographs in each age category. The number on top of the red portion of each bar indicates the percentage of females in each age category. The total numbers of each sex are shown at the top-right corner

Next, we made a list of patients who ever had assigned with any of the following international classification of diseases (ICD) codes: androgen insensitivity syndrome (ICD-9 259.5, ICD-10 E34.5), congenital adrenal hyperplasia (ICD-9 255.2, ICD-10 E25), chromosomal anomalies (ICD-9 758.6, 758.7, 758.8; ICD-10 Q96, Q97, Q98, Q99), and gender identity disorders (ICD-9 302.85, ICD-10 F64). The list contained 2189 patients, from which we found 444 left-hand radiographs. From the initially compiled dataset, we excluded 289 radiographs included in this list to avoid potential impacts of these conditions on sexual dimorphism in hand radiographs. The remaining 10,318 (5305 females and 5013 males) radiographs were used for training (7251), validation (1536), and testing (1531). For age-stratified analysis, we categorized the radiographs by age: by 1 year from 5 to 19 years old, 20 to 29, 30 to 39, 40 to 49, and 50 and older. The age and sex distribution of the subjects included in the final dataset is described in Fig. 1. The age ranged from 5 to 70 years with the median of 12. Lastly, we found 155 patients in the ICD-based list whose left-hand radiographs were available. Among these 155 patients, chart review by a physician board certified in internal medicine identified 44 patients who are confirmed to have the conditions.

### Data Preprocessing

**Fig. 2:**
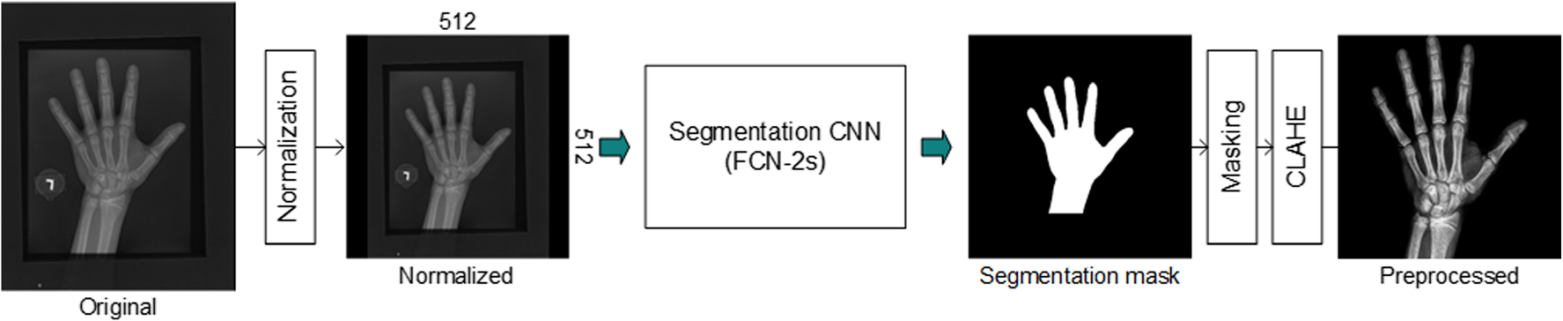
Data preprocessing pipeline. An overview of data preprocessing engine that normalizes radiographs to have a uniform size of 512 × 512 pixels, segments a region of the hand and wrist using a segmentation CNN, and enhances image contrast using contrast limited adaptive histogram equalization (CLAHE)

The radiographs varied considerably in intensity, contrast, image resolution, and existence of artifacts. To allow deep learning models to learn salient features, a preprocessing pipeline (Fig. 2) was implemented based on the previously developed module and modified by replacing the conventional detection CNN with a newly trained segmentation CNN [4]. The network architecture (FCN-2S) that performed best to segment skeletal muscle regions at the level of third lumbar (L3) vertebral body was trained and validated on the preliminarily compiled datasets for segmenting regions of the hand and wrist [4, 5]. The new segmentation CNN achieved the mean of intersection over union of 0.95. The preprocessing module first normalizes radiographs to have a uniform size (512 × 512 pixels) with preserving their aspect ratios, then segments a region of the hand and wrist and removes extraneous objects such as annotation markers and collimation. Subsequently, contrast limited adaptive histogram equalization (CLAHE) with default settings [6] was applied to the segmented and normalized images for contrast enhancement.

### Model Development and Network Training

The standardized images that went through the preprocessing engine were passed to a deep CNN (VGG16) [7] for bone sex classification. VGG16 is one of the CNNs validated in ImageNet Large Scale Visual Recognition Competition (ILSVRC) [8] for its decent classification performance. The CNN was pretrained on ImageNet [9] (a 1.28-M training images with 1000 class labels), modified by replacing the fully connected layers with a global average pooling [10], a fully connected, and a sigmoid layer, and then fine-tuned on our train dataset. We trained the classification and segmentation models for 100 epochs using a mini-batch stochastic gradient descent (SGD) with 0.9 Nesterov [11] momentum and a batch size of 64. A base learning rate of 10^−3^ and a weight decay of 5 × 10^−5^ were used for training the classification CNN, and a base learning rate of 10^−10^ and a weight decay of 10^−12^ were used for training the segmentation CNN. The base learning rates were decreased by a factor of 10 every 33 epochs for stable convergence of training loss function. The best models were selected based on validation losses. Keras (version 2.1.1) with a Tensorflow backend (version 1.3.0) were used as deep learning framework to develop models, and an NVIDIA Devbox (Santa Clara, CA) equipped with four TITAN X GPUs with 12 GB of memory per GPU was utilized to perform all experiments.

### Visualization

**Fig. 3:**
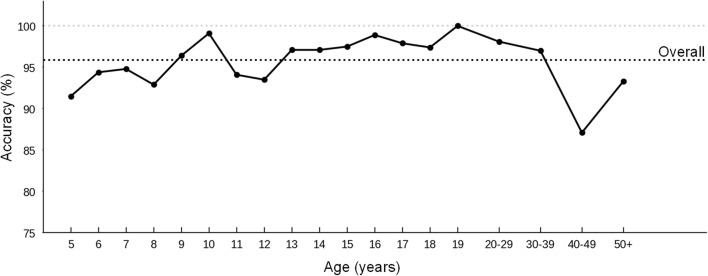
Age-stratified test accuracies. Test accuracies were shown as percent accuracy and stratified by age. The black dotted line indicates the overall accuracy across all 1531 radiographs in the test dataset

We investigated the intermediate features learned by the CNN using *t* distributed Stochastic Neighbor Embedding (*t*-SNE) [12]. Each test image was presented to the trained CNN to obtain the corresponding 512-dimensional features from the last convolutional layer. The high dimensional representation was converted into the 2-dimensional data, and the lower dimensional features were then visualized as shown in Fig. 3.

### Heat Map and Atlas Generation

Two visualization techniques were utilized to determine salient features that the model used for bone sex classification. First, class activation mapping [13] (CAM) technique was applied to the trained models to generate attention maps that highlight significant pixels for model predictions. Second, a set of training images most relevant to a given test case were retrieved from an atlas that consists of visual depictions of important features of each bone sex. The atlas was created by feeding all training images through the trained classification model, tracking all activation values of feature maps at the last convolutional layer, and keeping training images that caused highest activations on each feature map. During inference, gradients of a predicted output with regard to individual feature maps at the last convolutional layer were calculated via backpropagation, and the associated training images and attention maps with highest gradient were retrieved as the prediction basis of the model for a given case.

### Radiologist Evaluation

To test if human radiologists can see the sexual difference in hand radiographs, we evaluated radiologist performance on this task. Radiologist A, a diagnostic radiology board-certified physician with 15-year experience, and radiologist B in the third year of radiology residency, were blind-tested to predict sex from randomly selected 50 (24 females and 26 males) left-hand radiographs, for which the system predicted sex correctly with 100% confidence. DICOM files from which all patient information was removed were shown to the radiologists. The radiologists independently recorded their sex prediction for each case in a spreadsheet, and the result was compared with the phenotypic sex found in the electronic health record (EHR). After the feature description and attention localization process, the radiologists were tested again on the same 50 cases to assess if they learned any patterns during the process.

### Feature Description and Attention Localization

To find the features that distinguish the two sexes, the radiologists independently reviewed randomly selected radiographs with corresponding heat map and atlas, for which the model predicted sex correctly with 100% confidence. Radiologist A reviewed 70 cases (35 females and 35 males) and radiologist B reviewed 120 cases (60 females and 60 males) including the 70 cases reviewed by radiologist A. The cases used for the blind test were excluded when selecting these review cases. After the independent review, the two radiologists discussed their findings with each other. In addition, to localize the model attention, they checked the anatomical location of the heat maps of 50 cases using a customized spreadsheet. The localization was independently annotated, and radiologists were allowed to mark as many locations as they see. We counted the frequency of each location marked by either of the radiologists, separately for females and males. If two radiologists marked the same location in a case, the location was counted twice.

### Statistical Analysis

Stata version 14.2 (StataCorp, College Station, Texas, USA) was used for statistical analysis. Fisher’s exact test was used for the comparison of accuracy among age groups. The accuracy of the model and the radiologists were presented both as percent accuracy and Cohen’s kappa coefficient (*κ*) [14]. Cohen’s kappa was calculated using the formula *κ* = ( *p*_*o*_ − *p*_*e*_)/(1 − *p*_*e*_), where *p*_*o*_ = observed agreement among raters and *p*_*e*_ = expected agreement = $\frac{1}{N^2}{\sum}_k{n}_{k1}{n}_{k2}$ for categories *k*, number of items *N*, and *n*_ki_ the number of times rater *i* predicted category *k*.

## Results

### Model Accuracy

A deep learning model was developed and evaluated on the 1531 radiographs in the test dataset. The percent accuracies stratified by age groups are summarized in Fig. 3. The overall accuracy of the model on the test dataset was 95.9% and the *κ* = 0.918 (95% CI 0.898–0.938). The accuracies were not significantly different across all age groups (*p* = 0.232) as well as between females and males (*p* = 0.946).

### Radiologist Performance

On the initial test, radiologist A showed 58% accuracy (*κ* = 0.152, 95% CI = − 0.117–0.421). Radiologist B showed 46% accuracy (*κ* = − 0.077, 95% CI = − 0.351–0.198). On the second test after the feature description and attention localization process, the accuracy of radiologist A remained at 58% (*κ* = 0.160, 95% CI = − 0.113–0.433) and that of radiologist B was 42% (*κ* = − 0.164, 95% CI = − 0.437–0.109). The intra-reader agreement was 64% for radiologist A (*κ* = 0.280, 95% CI = 0.022–0.538) and 76% for radiologist B (*κ* = 0.523, 95% CI = 0.291–0.756). The results of radiologist performance assessment are presented in Table 1.

**Table 1:** Radiologist accuracies to predict sex from 50 randomly selected hand radiographs. Accuracies are presented as percent accuracy and Cohen’s kappa between radiologist prediction and phenotypic sex found in electronic health record. Intra-reader agreement measures agreement between the two test results by the same radiologist. Inter-reader agreement measures agreement between the two radiologists during the same test

	Initial test	Repeat test	Intra-reader agreement
Radiologist A	58% (*κ* = 0.152)	58% (*κ* = 0.160)	64% (*κ* = 0.280)
Radiologist B	46% (*κ* = − 0.077)	42% (*κ* = − 0.164)	76% (*κ* = 0.523)
Inter-reader agreement	52% (*κ* = 0.058)	48% (*κ* = − 0.040)	

### Feature Description and Attention Localization

**Fig. 4:**
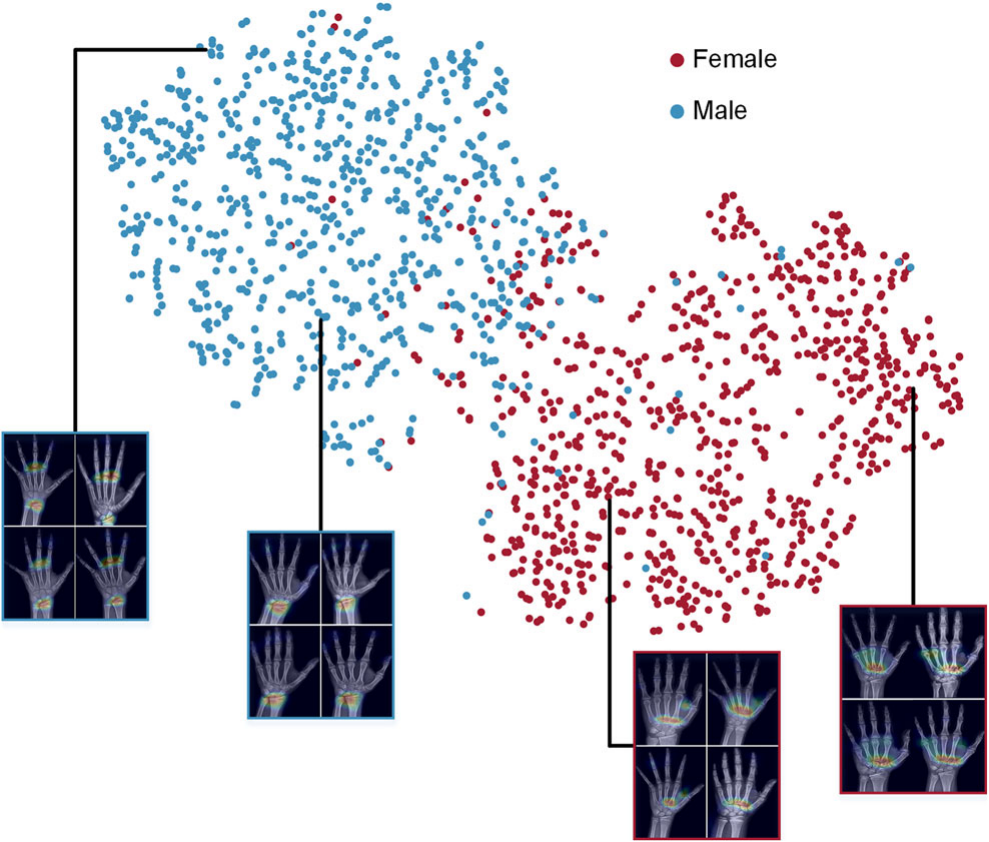
*t*-SNE visualization of the representations from the last convolutional layer of the model for bone sex classification. Here, we show how the algorithm clusters males and females. Radiographs with attention maps are linked to the corresponding points

The two radiologists did not find any consistent patterns that distinguish males and females in the hand radiographs. For the 50 cases for which the attention maps were annotated, each radiologist marked from 1 to 6 locations in each case. Collectively in all cases, there were 159 annotation counts for the 25 females and 103 annotation counts for the 25 males. In females, the second carpometacarpal joint was the most frequently localized (30 counts) followed by the third carpometacarpal joint (24 counts) and the thumb sesamoid (18 counts). In males, the most frequently localized region was the distal radioulnar joint (31 counts), the radial physis and epiphysis (13 counts), and the third metacarpophalangeal joint (9 counts). Figure 4 shows the representative images with overlaying heat maps in males and females as well as a *t*-SNE visualization.

### Preliminary Test on Selected Radiographs

Among the 44 patients selected by ICD code screening and chart review, 17 patients had congenital adrenal hyperplasia (9 females and 8 males), 6 females had Turner’s syndrome, 5 males had Klinefelter’s syndrome, 1 female had complete androgen insensitivity syndrome (CAIS) with 46, XY, 4 males had mixed gonadal dysgenesis, 8 individuals had female-to-male (FtM) transgender identity, and 3 individuals had male-to-female (MtF) identity. The overall accuracy of the model on the radiographs from these individuals was 77.8% (*κ* = 0.538, 95% CI = 0.288–0.788). Table 2 shows the agreement between model prediction and phenotypic sex.

**Table 2:** Agreement between algorithm prediction and phenotypic sex in a cohort with selected conditions. The ground truth for transgender individuals was set as the sex assigned at birth. Numbers in the parenthesis denotes (number of correctly predicted case)/(number of total case)

	Congenital adrenal hyperplasia	Chromosomal anomaly	Other primary hypogonadism	Transgender identity
Female	77.8% (7/9)	83.3% (5/6)^a^	100% (1/1)^c^	87.5% (7/8)^e^
Male	75.0% (6/8)	60.0% (3/5)^b^	75% (3/4)^d^	66.7% (2/3)^f^
Total	76.5% (13/17)	72.7% (8/11)	80% (4/5)	81.8% (9/11)

^a^Turner’s syndrome

^b^Klinefelter’s syndrome

^c^Complete androgen insensitivity syndrome

^d^Mixed gonadal dysgenesis

^e^Female-to-male transgender

^f^Male-to-female transgender

## Discussion

In this work, we developed an algorithm that accurately and reproducibly (*κ* > 0.9) identifies sex from hand radiographs and assessed the radiologists’ ability to do the same. Although it was generally agreed among radiologists that sex cannot be reliably determined by visual inspection of hand radiographs, we formally demonstrated this by showing very low agreement (*κ* < 0.2) between radiologist prediction and phenotypic sex even after a review session. The low inter-reader agreement (*κ* < 0.1) also indicates the near-randomness of sex prediction by radiologists. To the best of our knowledge, no previous research has shown that human radiologists cannot attain the performance of a machine learning algorithm for identifying a feature in radiographs. The results of our study show that radiographs contain more information than that is currently being utilized for clinical interpretation.

Previous studies about skeletal sexual dimorphism have shown differences between men and women. Men have bigger and stronger bones compared to women, and the difference is established mostly during puberty [15]. Based on such difference, automated sex estimation methods in adults have been developed using computed tomography (CT) scans and 3D imaging of the pelvis [16] and skull [17]. However, these methods were developed primarily for forensic anthropological analysis and use CT scans, limiting the more widespread use due to high radiation and cost. In hand and wrist, the most widely known feature is the digit ratio (2D:4D) that is frequently used as a biomarker for perinatal sex hormone exposure. However, despite numerous studies that investigated the subject, the association between digit ratio and perinatal sex hormone is still not confirmed [18]. In addition, there is substantial overlap between the distributions of digit ratio in males and females, thus any inference based on an individual’s digit ratio would be inaccurate [19, 20]. So far, the most prominent sexual difference in the skeletal structure of the hand and wrist is the size and volume of the bones [21]. Using this difference, a study that examined digital hand radiographs to construct a statistical classification model achieved an accuracy of 91% [22]. However, this study only included subjects that are over 18 years of age and requires measurement of 8 variables in the hand and wrist bones. This method not only requires the tedious process of measuring multiple points on radiographs but also is not applicable for children. We believe that our model is the first to classify sex using hand radiographs of all ages, regardless of the method.

Recent work that used deep learning as the primary method also showed the potential for detecting features that have not been established to be extracted from various medical images. In a study that used retinal fundus photographs to predict cardiovascular risk factors [2], the model accurately predicted gender, which has not been considered to be identifiable from retinal images. In another study, researchers showed that a machine learning approach can reliably predict cell nuclei, cell viability, cell type, and subcellular process type from transmitted light images [23]. These features require fluorescent labels for human scientists to detect. Although these studies did not investigate the capability of human experts to perform the same task, they also suggest that deep learning algorithms can be developed to find features that are not seen by humans.

In addition to showing the capability of seeing patterns not perceived by human experts, our model proposes a new indicator of skeletal gender, i.e., bone sex. It can be used to make more robust models for bone age assessment and other tasks that use sex as a key factor. Using this model, we can explore the association of bone sex and various clinical outcomes such as bone maturation patterns or development and prognosis of musculoskeletal diseases. This extends the utility of bone age radiographs beyond chronological age determination to potential assessment of sexual development or sex-determining hormone exposure. The persistent use of digit ratios to evaluate cancer risks [24, 25], development of mental disorders [26, 27], and sporting performance [28], despite its questionable accuracy for these tasks, underscores the need for such a biomarker. With further research that clarifies the factors associated with bone sex, especially in patients whose bone sex is discordant with their phenotypic sex, it could be used to detect early signs of certain conditions.

To explore the potential for the clinical implication of bone sex, we tested the model on a selected group of patients with a condition that could be associated with abnormal sexual development or disrupted sex hormone exposure. Numerous studies observed significant association between digit ratio with sex hormone levels, congenital adrenal hyperplasia, homosexuality [29], or transgender identity [30]. In addition, it is widely known that sex chromosome abnormality and congenital adrenal hyperplasia affect bone metabolism [31–33]. Although we could not draw a conclusion from this data because of the small sample size and possible selection bias, the lower accuracy compared to that in the test cohort implies the possibility that the discrepancy between model prediction and phenotypic sex reflects the altered skeletal development associated with these conditions. Further research in a well-defined cohort will provide more insight into the clinical use of bone sex.

One limitation of this study is that we only included left-hand radiographs to develop and validate the model. Although this study was focused more on the ability of a deep learning model to discover a pattern that has previously not been described, only including left-hand images could limit the use of the model in the clinical practice and other research studies. While it is the standard procedure to use the left hand for bone age assessment [34, 35], some studies suggest that sex differences in digit ratio are more profound in the right hand [36]. Further studies that include the right hand for testing and development will render a more comprehensive model to be clinically used.

An additional limitation is our inability to identify radiographic features that are sex-specific, despite the availability of CNN heat maps and atlas. This is a universal issue with deep learning as the inner-workings of the resulting algorithm are not completely understood. We used the heat maps and the atlas to assist the radiologists in identifying distinguishing features, to no avail. We believe this is an important result of our work that highlights the power of artificial intelligence to go beyond the limits of human visual perception. Further work is needed to identify sex-specific radiographic features and teach them to human users. To use the additional information obtained by using this innovative technology, clinicians must be cautious and understand how deep learning algorithms work.

## Conclusion

We developed a deep learning model that distinguishes males from females based on hand and wrist radiographs, a task that human radiologists failed to reproduce. The current study shows that deep learning can be used to identify patterns that are beyond human perception.
